# External validation of the REDUCTION clinical decision aids for ruling out fractures in patients with midfacial and mandibular injuries in the emergency department: a protocol for a prospective multi-centre cohort study

**DOI:** 10.1186/s13049-026-01548-x

**Published:** 2026-01-16

**Authors:** Pieter Date van der Zaag, Romke Rozema, Inge H. F. Reininga, Baucke van Minnen

**Affiliations:** 1 Department of Oral and Maxillofacial Surgery, University Medical Center Groningen, University of Groningen, Groningen, The Netherlands; 2 Department of Surgery, University Medical Center Groningen, University of Groningen, Groningen, The Netherlands; 3 Department of Trauma Surgery, University Medical Center Groningen, University of Groningen, Groningen, The Netherlands

**Keywords:** Clinical decision aid, Computed tomography, Diagnostic accuracy, Maxillofacial injury, Maxillofacial fractures, Physical examination findings, Emergency department

## Abstract

**Background:**

Computed Tomography (CT) is the gold standard for diagnosing midfacial and mandibular fractures in emergency department (ED) patients. However, its increasing availability has led to more frequent use, resulting in higher healthcare costs and unnecessary radiation exposure. Although various clinical parameters can assist in diagnosis of maxillofacial fractures, none are reliable enough to rule them out individually. To support clinical decision-making, and to reduce unnecessary imaging, the REDUCTION studies developed four decision aids based on combinations of clinical parameters. The aim is to rule out: (I) midfacial fractures, (II) midfacial fractures requiring active treatment, (III) mandibular fractures, and (IV) mandibular fractures requiring active treatment. Before being implemented, these aids must undergo external validation.

**Methods:**

A prospective multi-centre validation study will be conducted in six hospitals in the Netherlands. Patients presenting at the ED with midfacial and/or mandibular injury will undergo a standardized physical examination based on the parameters included in the decision aids. The X-ray images will be reviewed to assess for fractures, and electronic patient records will be analyzed to determine whether fracture treatment was active or conservative. The diagnostic accuracy of each decision aid will be determined by calculating their sensitivity, specificity, and negative predictive value.

**Discussion:**

The REDUCTION validation trial will assess whether the developed clinical decision aids can be used safely to reduce unnecessary CT scans in patients with midfacial or mandibular injuries at the ED. If accurate, these decision aids could be integrated into emergency care to support decision-making, leading to fewer CT scans, lower radiation exposure, reduced healthcare costs, and safe, efficient management of maxillofacial injury.

**Trial registration:**

ICTRP: NL-OMON56277.

## Background

Currently, computed tomography (CT) is the gold standard for the diagnosis of midfacial and mandibular fractures at the emergency department (ED). The easy availability of CT scans has led to an exponential increase in their use for patients with suspected midfacial and mandibular fractures. This, in turn, has led to an increase in costs and radiation exposure within this patient population. Regarding both midfacial and mandibular fractures, specific clinical parameters have shown to contribute, but not decisively, to ruling out fractures [1]. Testing these specific clinical parameters in a set, for example in a clinical decision aid, could assist in deciding whether to perform imaging for diagnosing or ruling out a fracture and/or for predicting the required treatment [2, 3].

Thus, a decision aid for ruling out midfacial or mandibular fractures could reduce the amount of unnecessary imaging of patients with injuries to the midface and mandible and, thereby, reduce costs and radiation exposure. Hence, the REDUCTION study group was established as a multidisciplinary collaboration between the emergency medicine, radiology, trauma surgery and oral and maxillofacial surgery departments to reduce unnecessary imaging, resulting in the REDUCTION I and II studies [2, 3]. The intent of these decision aids is to reduce CT imaging by enabling emergency department clinicians to rule out fractures when all parameters are absent, given their high negative predictive value, while the CT remains available when clinical suspicion persists or when needed for diagnostic or treatment planning purposes.

It is important to note that not all maxillofacial fractures require active treatment i.e. splinting, intermaxillary fixation or surgical repositioning. Fractures can often be managed conservatively with adequate analgesics, avoidance of nose blowing and a soft non-chewing diet [3]. Therefore, the REDUCTION studies distinguished between fractures, and those requiring active treatment. Four decision aids were developed based on clinical parameters to rule out: I) the presence of midfacial fractures, II) the presence of midfacial fractures that require active treatment, III) the presence of mandibular fractures, and IV) the presence of mandibular fractures that require active treatment [2, 3].

The midfacial injury decision aid I consists of the following clinical parameters: peri-orbital haematoma, epistaxis, ocular movement limitation, infra-orbital nerve paraesthesia, palpable step-off and tooth mobility or avulsion. It showed a sensitivity of 89.7 (95%CI 86.0–92.5), a specificity of 42.6 (95%CI 38.0–47.4), and a negative predictive value of 83.9% (95%CI 78.4–88.2) for ruling out midfacial fractures [2]. The midfacial injury decision aid II consists of the following clinical parameters: facial depression, epistaxis, ocular movement limitation, palpable step-off, objective malocclusion and tooth mobility or avulsion. It showed a sensitivity of 97.3 (95%CI 90.7–99.3), a specificity of 38.6 (95%CI 35.0–42.3), and a negative predictive value of 99.3 (95%CI 97.3–99.8) for ruling out midfacial fractures that require active treatment [3].

The mandibular injury decision aid III consists of the following clinical parameters: the angular compression test, axial chin pressure test, objective malocclusion, tooth mobility or avulsion and the tongue blade bite test. It showed a sensitivity of 98.5 (95%CI 91.9–99.7), a specificity of 34.6 (95%CI 28.5–41.2), and a negative predictive value of 98.7% (95%CI 92.8–99.8) for ruling out mandibular fractures [2]. The mandibular injury decision aid IV consists of the following clinical parameters: mouth opening limitation, jaw movement pain, objective malocclusion and tooth mobility or avulsion. It showed a sensitivity of 100.0 (95%CI 90.6–100.0), a specificity of 39.1 (95%CI 33.2–45.4), and a negative predictive value of 100.0 (95%CI 96.1 100.0) for ruling out mandibular fractures that require active treatment [3].

The initial analysis showed a possible 5–15% proportional reduction in maxillofacial CT scans in the ED, thereby reducing unnecessary scans. However, the external validity of these clinical decision aids needs to be established first. External validation involves testing the diagnostic accuracy of the clinical decision aids in a different patient population than the one in which they were developed. If the decision aids achieve the desired diagnostic accuracy in a different population, they can be incorporated into routine ED clinical practice.

## Methods

### Objective

The primary objective of the REDUCTION Validation Trial is to validate the clinical decision aids externally that were developed in the REDUCTION I & II studies of patients presenting with midfacial and/or mandibular injuries in the emergency department.

### Trial design

A prospective multicentre cohort study. The Medical Ethics Committee (METc) of the University Medical Center Groningen (UMCG) approved the clinical trial and its amendment (METc code 2023/267). Local feasibility has been approved by all the external hospitals participating in the study. The study is conducted in accordance with the Declaration of Helsinki and the FEDERA code of conduct. The study was registered with the International Clinical Trials Registry Platform (ICTRP) under the reference number NL-OMON56277.

### Study setting

The study will be led by the Department of Oral and Maxillofacial Surgery (OMF) of the UMCG, and data will be collected from the ED patients of the UMCG, Nij smellinghe Ziekenhuis, Isala klinieken Zwolle & Meppel, Frisius Medical Centre Leeuwarden and Ommelander Ziekenhuis Groningen, in the Netherlands. The participating hospital EDs cover the entire hospital trauma level ranges (levels 1–3).

### Eligibility criteria

All consecutive patients aged 18 years and older presenting with midfacial and/or mandibular injury at the participating hospital EDs are eligible for inclusion if the following conditions are met:

- Inclusion criteria:
  - Visible injuries to the face or in the oral cavity.
  - A trauma mechanism capable of causing facial fractures, e.g. violence, falls, traffic accidents, etc.
  - An anamnesis or case history that clearly indicates a facial trauma.
  - Patients aged ≥ 18 years.

- Exclusion criteria:
  - Patients with a non-energetic maxillofacial injury i.e. cutting wounds or spontaneous epistaxis.
  - Patients who are not admitted for the first time with maxillofacial injury within the inclusion period.
  - Patients who have declined access to medical records and are registered in the objection register.
  - Patients referred to another participating hospital after having already been included in the primary hospital’s study.

### Interventions

#### Midfacial injury

Patients with midfacial injuries will undergo a physical examination and CT imaging at the ED, or a Cone Beam Computed Tomography (CBCT) or a at the OMF outpatient clinic. Decision aid I predicts the presence of midfacial fractures, while Decision aid II predicts the presence of midfacial fractures requiring active treatment [2, 3]. As there is an overlap in both decision aids’ clinical parameters, they will be combined into a single physical examination of midfacial injuries in the external REDUCTION-Validation Trial (see Table 1). The specific definitions are described in Appendix 1.

**Table 1:** Midfacial injury physical examination parameters

Clinical parameters	Decision aid I	Decision aid II	REDUCTION-validation physical examination
1. Peri-orbital haematoma	X		X
2. Epistaxis	X	X	X
3. Ocular movement limitation	X	X	X
4. Paraesthesia infra-orbital nerve	X		X
5. Palpable bony step-off	X	X	X
6. Mobility or avulsion of teeth (18–28)	X	X	X
7. Facial depression		X	X
8. Objective malocclusion		X	X

The physical examination of midfacial injury patients will therefore consist of checking the presence of eight clinical parameters: facial depression, peri-orbital haematoma, epistaxis, ocular movement limitation, infra-orbital nerve paraesthesia, palpable bony step-off, objective malocclusion and tooth mobility or avulsion. Assessments of these clinical parameters will be scored as either present or absent, with an additional option of "not testable" if a clinical parameter cannot be assessed. For example, if there is severe swelling and/or haematoma, the patient is intubated, or if the patient's level of consciousness does not allow for active patient instruction.

### Mandibular injury

The intervention for patients with mandibular injuries consists of a physical examination and CT imaging at the ED, a CBCT or an orthopantomography (OPT) at the OMF outpatient clinic. Decision aid III predicts the presence of mandibular fractures, while Decision aid IV predicts the presence of mandibular fractures requiring active treatment [2, 3]. As both decision aids’ clinical parameters overlap, they will be combined into a single physical examination for mandibular injuries in the external REDUCTION-Validation Trial (see Table 2). The specific definitions are described in Appendix 2.

**Table 2:** Mandibular injury physical examination parameters

Clinical parameters	Decision aid III	Decision aid IV	REDUCTION-validation physical examination
1. Angular compression test	X		X
2. Axial chin pressure test	X		X
3. Objective malocclusion	X	X	X
4. The tongue blade bite test	X		X
5. Tooth mobility or avulsion (38–48)	X	X	X
6. Mouth opening limitations		X	X
7. Jaw movement pain		X	X

The physical examination of mandibular injury patients will therefore consist of checking the presence or absence of seven clinical parameters: angular compression test, axial chin-pressure test, objective malocclusion, the tongue blade bite test, mouth opening limitations, jaw movement pain and tooth mobility or avulsion. Assessments of these clinical parameters will be scored as either present or absent, with an additional option of "not testable" if a clinical parameter cannot be assessed.

### Radiological imaging

Given that the endpoint of this study is verifying the presence or absence of a maxillofacial fracture, all participating patients with a midfacial or mandibular injury will require maxillofacial imaging. In general, CT is considered to be the gold standard for establishing the presence or absence of a maxillofacial fracture.

Accordingly most of the patients presenting with a maxillofacial injury at the ED will undergo CT imaging according to the standard care protocols. This will be based on maxillofacial injury severity or the presence of concomitant head or neck trauma, and to ensure timely detection and management of fractures in high-risk cases. However, a subset of patients will not meet the criteria for immediate CT imaging at the ED, but may still harbour maxillofacial fractures requiring confirmation.

Besides CT, two other imaging modalities used for diagnosing maxillofacial fractures are the CBCT and the OPT. While a maxillofacial CT scan provides the highest image quality, it involves a relatively high radiation dose, which—in accordance with the ALARA (As Low As Reasonably Achievable) principle—is not desirable in patients with low clinical suspicion of maxillofacial fracture [4]. The OPT, a two-dimensional technique, is frequently used as an initial modality for suspected mandibular fractures due to its low radiation dose [5]. However, it lacks the three-dimensional capability required to assess displacement or to evaluate midfacial fractures. Therefore, CBCT is the preferred imaging modality for this study to rule out maxillofacial fractures in patients initially seen in the ED who did not receive a CT scan [6–8].

The ED patients, who did not receive a CT scan, will receive an information package about the REDUCTION-V trial, including an informed consent (IC) form and details about participating in the study. Patients expressing an interest will be contacted within a week to schedule an appointment at the OMF clinic within a month. This timeframe balances the need for informed decision-making, with the opportunity to detect and treat missed maxillofacial fractures.

**Fig. 1:**
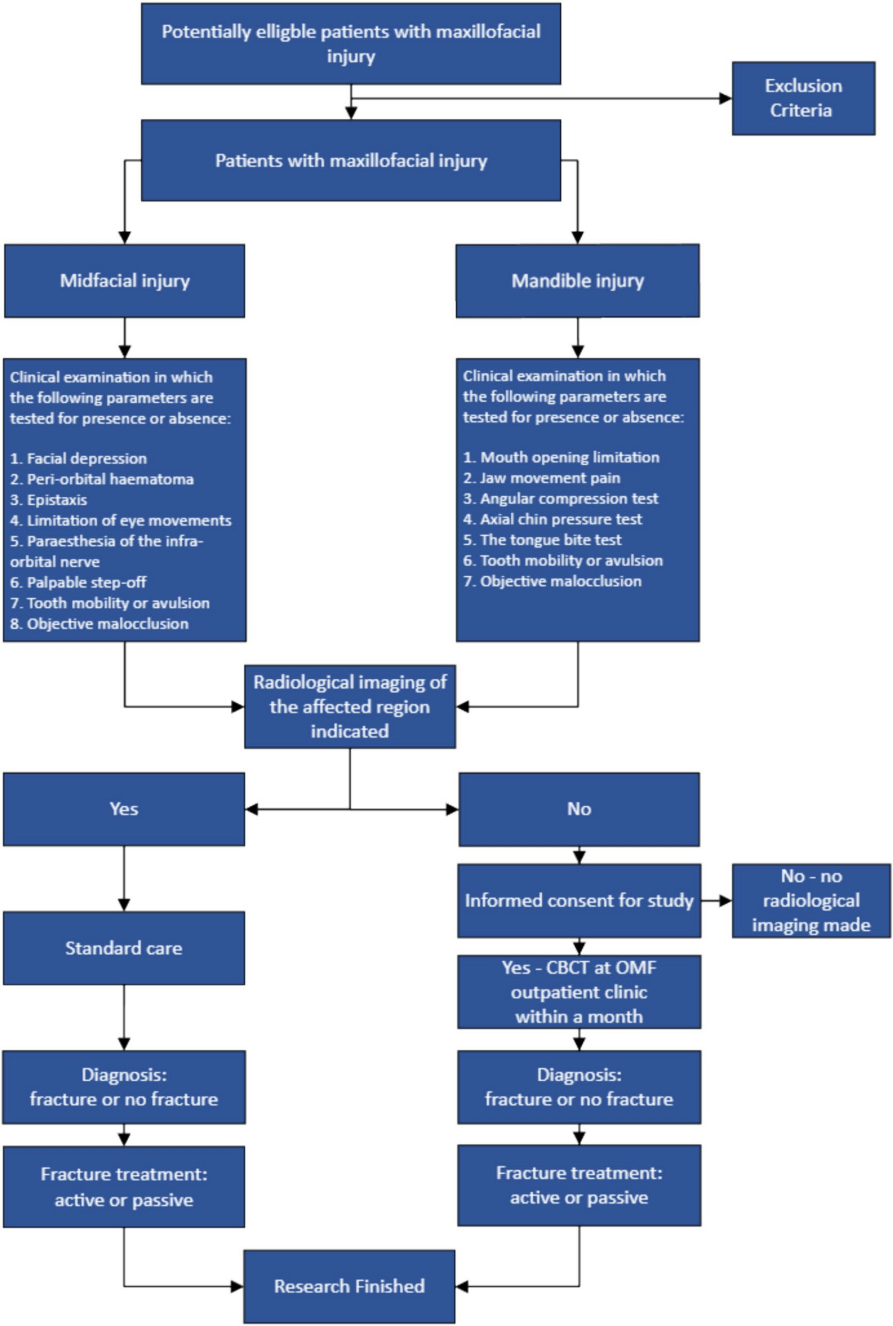
Flowchart of participating maxillofacial injury patients

At the OMF clinic, the patients will attend a consultation with an OMF surgeon specialized in trauma who will inform them of the study. Any remaining questions can be addressed, and the IC form can be signed. A CBCT scan will then be performed to definitively rule out fractures. However, if a maxillofacial fracture is identified, the OMF surgeon will initiate appropriate treatment immediately, according to the standard care (see Fig. 1).

Moreover, a small proportion of patients presenting solely with a maxillofacial injury may be referred directly to the OMF outpatient clinic by a general practitioner or dentist, rather than to the ED. In these cases, CBCT is used to evaluate suspected midfacial fractures, while in selection of cases an OPT is performed when a mandibular fracture is suspected. In accordance with the ALARA and standard care, these patients with mandibular injury will not receive an additional CBCT scan unless medically necessary.

### Outcomes

The first primary outcome of the study is the sensitivity, specificity, and negative predictive value of the decision aids I and III in predicting and ruling out the presence of either a midfacial or mandibular fracture, compared to radiographic imaging. Midfacial fractures are defined as any fracture of the frontal sinus, orbital rim and walls, maxillary sinus, zygomaticomaxillary complex, nasoorbitoethmoid (NOE) complex, nasal bone, Le Fort I, II, III complex, and maxillary dentoalveolar complex [9]. Mandibular fractures are defined as any fracture of the symphyseal, parasymphyseal, corpus, angular, ramus, coronoid and condylar process, and fractures of the mandibular dentoalveolar complex [10].

The second primary outcome of the study is the sensitivity, specificity and negative predictive value of the decision aids II and IV in predicting and ruling out the presence of either a midfacial or mandibular fracture requiring active treatment, compared with the decision on how to treat the midfacial or mandibular fracture at the ED. Conservative treatment includes adequate analgesics, avoidance of nose blowing or holding the nose when sneezing, a soft non-chewing diet, and watchful observation. Active treatment is divided into closed or open treatment. Closed treatment includes reduction of nasal fractures under local anaesthesia, nasal packing, intermaxillary fixation, rigid and flexible splinting or appliances for dental injury. Open treatment includes any surgical intervention in which the patient undergoes open reduction and internal fixation in an operation theatre. Decisions will be made according to the standard care, in agreement with the treatment protocols of the Dutch Society of Oral and Maxillofacial Surgery (NVMKA) or Dutch Association of Otorhinolaryngology and Head & Neck Surgery (NVKNO) when consulted within the inclusion period [11].

The secondary study outcomes are: Maxillofacial injury severity based on the Facial Injury Severity Score (FISS) [12]. Any maxillofacial fracture or laceration longer than 10 cm is scored from 1 to 6 on the FISS, and the sum of all scored fractures or lacerations gives an indication of the overall severity of the sustained maxillofacial injury. Overall injury severity is based on the Abbreviated Injury Scale (AIS) and the Injury Severity Score (ISS) [13]. The AIS is a 6-point scale, with 1 being the least severe injury and 6 being the most severe, with the injury resulting in death. The AIS is used to calculate the Injury Severity Score (ISS) as a measure of overall injury severity. Also, the source of referral, mechanism of injury, age, sex, reported alcohol use, state of consciousness in accordance with the Glasgow Coma Score, status of intubation and sedation, presence of dental injury, edentulous status and skull fractures will be registered.

### Sample size

The results of this study will be presented in contingency tables. We do not expect any difference in the number of maxillofacial injuries, fractures or in improved predictive value of the decision aids compared to the initial REDUCTION trial. In the initial REDUCTION trial, 993 patients were included between May 2018 and October 2019. According to the literature, the rule-of-thumb of a sample size of at least 50 events can be applied to studies regarding the external validation of a clinical prediction model [14, 15]. Considering the assumption that the case-mix will be comparable to that of the initial REDUCTION trial, an overall sample size of N = 1200 will be sufficient to include at least 50 patients with an event i.e. the predicted outcome of the specific clinical decision aid – in each subgroup regarding the four clinical decision aids. To account for improperly completed patient forms, missing data, and dropouts in the CBCT subgroup, we assume there should be a margin of 100 patients. Accordingly, we aim to enrol 1300 patients with maxillofacial injuries in 24 months.

### Recruitment

Patients will be recruited at the ED of the participating centres. All consecutive patients with a maxillofacial injury aged 18 years or older are eligible for inclusion. To meet the increased target sample size compared to the previous REDUCTION trial, two additional hospitals — Frisius MC Leeuwarden and Ommelander Ziekenhuis Groningen, the Netherlands — have been added to expand the recruitment pool [2]. The enrolment will span a period of two years, compared to the one and a half years of the previous trial.

### Consent or assent

No explicit informed consent is required from the majority of patients. These primary subgroup patients will undergo a clinical examination and CT imaging as part of the standard care at the ED based on clinical indications such as injury severity or concomitant head or neck trauma. The Medical Ethics Review Committee (METc UMCG) has granted permission to use the data from these patients without informed consent, provided that the local opt-out register (bezwaarregister) is checked. To respect the patients’ privacy and autonomy, inclusion in the study will occur at least one month after the initial ED visit, ensuring they have had the opportunity to object to the secondary use of their data.

Regarding the smaller subset of patients—those with a low suspicion of midfacial or mandibular fractures, and who accordingly would not receive CT imaging as part of the standard care—a CBCT scan may be required as part of the study protocol. These secondary subgroup patients will receive an information package at the ED, including study details and a sample IC form. If the patient expresses interest, the OMF surgery team will contact them within a week to schedule a consultation at the OMF outpatient clinic within a month. During this visit, a trained and qualified member of the OMF team will answer any remaining questions and obtain written informed consent before proceeding with the CBCT scan.

### Data collection methods

All eligible patients will undergo a physical examination to assess the eight midfacial region and/or seven mandibular region parameters. According to the standard care protocol, the examinations will be performed by emergency physicians, surgeons, OMF surgeons or resident physicians from these specialties. Findings regarding each clinical parameter will be documented during the primary or secondary assessment and categorized as absent, present, or not assessable. To maintain objectivity, physical examinations will be conducted without any knowledge of the radiological imaging outcomes, unless immediate medical intervention is required. All these findings will be recorded in an online case report form (CRF) by the attending physician.

The primary outcome will be the sensitivity, specificity, and negative predictive value of the decision aids in predicting or ruling out the presence of a midfacial or mandibular fracture. Fractures will be diagnosed via CT, CBCT or OPT. The CT scans will be assessed by radiologists, while the CBCT and OPT will be evaluated by oral and maxillofacial surgeons. The results of the physical examination will be assessed with the intention of being blinded to radiological interpretations. However, complete blinding may not always be achievable due to standard clinical practice. Fracture classification will be conducted by a board-certified oral and maxillofacial surgeon. These findings will be initially recorded in the electronic patient files. Data will be collected from the patients’ files and will be recorded in an online CRF by the coordinating researcher.

### Data management

This study will be conducted in accordance with the General Data Protection Regulation (GDPR) and the Dutch GDPR execution act (UAVG). All the data obtained in this study will be handled with utmost care and confidentiality. All patient information regarding demographics and addresses will be kept safe in the electronic patient file (EPD) of the UMCG. Data gathered from the ED and follow-up are part of the standard of care and are therefore registered in the EPD, except for the CBCT data from the OMF outpatient clinic. Digitally recorded data on outcome measures will be stored in REDCap, a web-based clinical trial management system distributed by the UMCG [16, 17]. All patient data registered in the EPD, which are related to the study parameters, will then be registered in a CRF via the REDCap system, including interpretations of the images from the CT, CBCT or OPT. Other data processed for the study will be stored digitally on a secure online research-drive of the UMCG which makes automatic back-ups every night in CORSA, a back-up system of the OMF surgery department in the UMCG. This digital drive will only be accessible after a secured login by the researchers of this study and the research coordinator of the department of OMF surgery of the UMCG.

### Statistical methods

Categorical variables will be reported as frequencies and percentages. Normally distributed variables will be reported as means and standard deviations, and variables with a non-normal distribution will be reported as median and inter quartile range.

The results of the presence of maxillofacial fractures will be expressed as a confusion matrix, showing the presence of midfacial and mandibular fractures according to CT, CBCT or OPT imaging, as well as the prediction of fractures according to the REDUCTION I clinical decision aids. In accordance with the REDUCTION I study, the absence of clinical parameters will be listed as 'negative', whereas the presence and the not testable clinical parameters will be listed as 'positive' findings [2]. Therefore, for edentulous patients, classifying the tongue blade bite test, objective malocclusion, and tooth mobility or avulsion as non-testable would result in a ‘positive finding’. This would automatically render the clinical decision aid positive in all edentulous patients and influence diagnostic accuracy. To prevent this, these clinical signs will be recorded as absent in edentulous patients.

The diagnostic accuracy outcomes will be presented as the prevalence, pre-test probability, sensitivity, specificity, positive predictive value (PPV), negative predictive value (NPV), positive likelihood ratio (LR +) and negative likelihood ratio (LR –).

The results of the presence of maxillofacial fractures that require active treatment will be expressed as a confusion matrix, including the presence of midfacial and mandibular fractures that require active treatment according to the standard care, and in agreement with the treatment protocols, as well as the predicted fractures needing treatment according to the REDUCTION II clinical decision aids. In accordance with the REDUCTION II study, the absence of clinical parameters will be listed as 'negative', whereas the presence and the not testable clinical parameters will be listed as 'positive' findings [3]. In edentulous patients, objective malocclusion and tooth mobility will be recorded as absent. The diagnostic accuracy outcomes will be presented as the prevalence, pre-test probability, sensitivity, specificity, PPV, NPV, LR + and LR –.

## Discussion

The REDUCTION validation trial will externally validate the clinical decision aids developed in the REDUCTION study in a new patient population. This will allow for assessment of their diagnostic accuracy and determine whether these aids can safely reduce the number of unnecessary CT scans in patients with midfacial or mandibular injuries at the emergency department. Therefore, the REDUCTION decision aids should be considered as triage tools that guide ED physicians on when CT imaging can safely be deferred without replacing a specialist's assessment or the use of CT when clinically indicated. If proven accurate, the decision aids could be integrated into emergency care to support clinical decision-making. This could result in fewer CT scans, reduced radiation exposure, lower healthcare costs, and more efficient and safer management of maxillofacial injuries.

Preliminary analyses suggest a potential proportional reduction of 5–15% in the number of maxillofacial CT scans performed in the ED. While this reduction may seem modest, the high global volume of CT imaging in emergency care implies that even small decreases could have a significant overall impact [18–20]. As part of the validation process, we will critically assess the following: 1, whether any fractures missed by the clinical decision aid had significant clinical consequences and 2, whether the decision aids truly reduce the need for dedicated maxillofacial CT imaging, or if the diagnosis was established through concurrent CT scans of the cerebrum or neck. This could potentially limit the actual reduction in maxillofacial CT imaging. This distinction is crucial for realistically evaluating the clinical safety and economic impact of implementation.

It is possible that MRI will be suitable for diagnosis of fractures in the ED setting in the future [21]. This will solve the issue of radiation, but will still be costly and time consuming. Therefore, clinical decision aids to rule out fractures will be of lasting value.

For many reasons it is beneficial to keep patients out of the hospital. Therefore, we aim for future implementation of these decision aids in primary care settings, such as out-of-hours general practitioner (GP) centres, is. Since CT imaging is unavailable in these settings, the decision aids could help GPs identify low-risk patients and avoid unnecessary ED referrals. However, since this is a different patient population than that of the ED, a follow-up validation study would be required.

## Data Availability

The datasets generated during the current study are not publicly available yet as the study is still ongoing and not all data have been anonymized. Once the study is completed and full anonymization has been ensured, the data may be made available from the corresponding author upon reasonable request.

## Appendix 1

### Definition of the midface physical examination clinical parameters

**Table:**

Facial depression	Unilateral flattening or depression of the malar eminence, cheek or zygomaticomaxillary complex. A “*yes”* is scored if there is unilateral facial depression. A “*no*” is scored if there is no unilateral facial depression. A “*not testable”* is scored if unilateral facial depression cannot be assessed, for example in situations where a physical examination is not possible due severe swelling or excessive pain during palpation.
Peri-orbital haematoma	Any haematoma localized within or around the orbital or zygomaticomaxillary area that is not defined as raccoon eyes. A “*yes*” is scored if there is a peri-orbital haematoma. A “*no*” is scored if there is no peri-orbital haematoma. A “*not testable*” is scored if the presence of a peri-orbital haematoma cannot be assessed.
Epistaxis	A unilateral or bilateral active or passed nosebleed. A “*yes*” is scored for cases with (past) epistaxis. A “*no*” is scored if there is no (past) epistaxis. A “*not testable*” is scored if the presence of epistaxis cannot be assessed.
Ocular movement limitation	Any restricted gazing or limitation of the eye movements in any direction. A “*yes*” is scored if there are ocular movement limitations. A “*no*” is scored if there are no ocular movement limitations. A “*not testable*” is scored if ocular movements cannot be assessed, for example in case of severe swelling and/or haematoma, or if the patient’s state of consciousness hinders active instructing.
Infra-orbital nerve paraesthesia	Any numbness, change or loss of sensation of the infraorbital nerve innervation area (e.g., the lower eyelid, nasal vestibule, part of the cheek, the upper lip, upper incisor, canine and premolars). A “*yes*” is scored if there is infra-orbital nerve paraesthesia. A “*no*” is scored if there is no infra-orbital nerve paraesthesia. A “*not testable*” is scored if the presence of infra-orbital nerve paraesthesia cannot be assessed, for example if the patient’s state of consciousness hinders communication.
Objective malocclusion	Traumatic misalignment or incorrect relation between the teeth of the two dental arches as objectively identified by the assessor during the intra-oral examination. A “*yes*” is scored if there is malocclusion. A “*no*” is scored if there is no malocclusion. A “*not testable*” is scored if the occlusion cannot be assessed, for example if the patient has dentures, is edentulous or if the patient is intubated and sedated.
Tooth mobility or avulsion	Mobility or avulsion of any maxillary tooth elements (e.g., first (11-18) and second (21-28) quadrant tooth elements). A “*yes*” is scored in case of tooth mobility or avulsion. A “*no*” is scored if there is no tooth mobility or avulsion. A “*not testable*” is scored if the presence of tooth mobility or avulsion cannot be assessed, for example if the patient cannot be suitably instructed.
Palpable step-off	The presence of a bony step-off, step defect or discontinuity found when palpating any of the following midfacial anatomical landmarks: (1) zygomatic arch, (2) infra-orbital rim, (3) supra and lateral orbital rim, (4) nasal bridge and (5) zygomaticalveolar crest intra orally. A “*yes*” is scored if there is a palpable step-off. A “*no*” is scored if there is no palpable step-off. A “*not testable*” is scored if the presence of a palpable step-off cannot be assessed, for example in case where swelling and/or haematoma hinders full palpable examination of the midface.

## Appendix 2

### Definition of the mandibula physical examination clinical parameters

**Table:**

Jaw movement pain	The presence of pain during opening, protrusion or lateral movement(s) of the mandible. A “*yes*” is scored if there is pain with jaw movement(s). A “*no*” is scored if there is full range movement of the jaw without pain. A “*not testable*” is scored if pain during jaw movements cannot be tested, for example when the patient’s state of consciousness hinders adequate communication.
Mouth opening limitations	The reported inability to open the mouth fully or there is a measured restriction of the mouth opening of 35 mm or less while opening the mandible. A “*yes*” is scored for limited mouth opening cases. A “*no*” is scored if there are no mouth opening limitations. A “*not testable*” is scored if the mouth opening limitations cannot be assessed, for example when the patient’s state of consciousness hinders adequate communication.
Tooth mobility or avulsion	Mobility or avulsion of any mandibular tooth element (e.g., third (31-38) and fourth (41-48) quadrant tooth elements). A “*yes*” is scored in case of tooth mobility or avulsion. A “*no*” is scored if there is no tooth mobility or avulsion. A “*not testable*” is scored if the presence of tooth mobility or avulsion cannot be assessed, for example if the patient cannot be suitably instructed.
Objective malocclusion	Traumatic misalignment or incorrect relation between the teeth of the two dental arches, as identified objectively by the assessor during the intra-oral examination assessment. A “*yes*” is scored if there is malocclusion. A “*no*” is scored if there is no malocclusion. A “*not testable*” is scored if the occlusion cannot be assessed, for example if the patient has dentures, the patient is edentulous or if the patient is intubated and sedated.
Angular compression test pain	Noteworthy presence of pain complaints in the symphyseal or para-symphyseal region induced by simultaneous bilateral pressure of the mandibular angle, also known as the angular compression test. A “*yes*” is scored in case of noteworthy presence of pain during the angular compression test. A “*no*“ is scored if there is no pain during the angular compression test. A “*not testable*” is scored when the angular compression test cannot be conducted, for example if communication with the patient is not possible.
Axial chin pressure test pain	Noteworthy unilateral or bilateral presence of pain complaints in the condylar or temporomandibular region induced by axial pressure on the chin. A “*yes*” is scored in case of noteworthy pain during the axial chin pressure test. A “*no*” is scored if there is no pain during the axial chin pressure test. A “*not testable*” is scored when the axial chin pressure test cannot be conducted, for example if communication with the patient is not possible.
Tongue blade bite test	The patient’s ability to maintain bilateral intermaxillary fixation of a uniform wooden spatula or tongue depressor (150 x 18 x 1.6 mm) while it is broken by medial rotation by the assessor. A “*yes*” is scored if the patient is unable to maintain bilateral fixation of the wooden spatula during the tongue blade bite test. A “*no”* is scored if the patient is able to maintain bilateral fixation of the wooden spatula during the tongue blade bite test. A “*not testable*” is scored if the tongue blade bite test cannot be conducted, for example in a situation where the patient cannot be instructed.

## Abbreviations

ED: Emergency Department
CT: Computed Tomography
METc: The Medical Ethics Committee
UMCG: University Medical Center Groningen
ICTRP: International Clinical Trials Registry Platform
OMF: Oral and Maxillofacial Surgery
CBCT: Cone Beam Computed Tomography
OPT: Orthopantomography
ALARA: As Low As Reasonably Achievable
IC: Informed Consent
NVMKA: Dutch Society of Oral and Maxillofacial Surgery
NVKNO: Dutch Association of Otorhinolaryngology and Head & Neck Surgery
FISS: Facial Injury Severity Score
AIS: Abbreviated Injury Scale
ISS: Injury Severity Score
CRF: Online Case Report Form
GDPR: General Data Protection Regulation
UAVG: Dutch GDPR execution act
EPD: Electronic patient file
PPV: Positive predictive value
NPV: Negative predictive value
LR +: Positive likelihood ratio
LR –: Negative likelihood ratio
GP: General practitioner
